# 7-(2-Chloro­phen­yl)-2,6,9-trimethyl­dibenzo[*b*,*h*][1,6]naphthyridine

**DOI:** 10.1107/S1600536810030576

**Published:** 2010-08-28

**Authors:** K. N. Vennila, K. Prabha, M. Manoj, K.J. Rajendra Prasad, D. Velmurugan

**Affiliations:** a Centre of Advanced Study in Crystallography and Biophysics, University of Madras, Guindy Campus, Chennai 600 025, India; b Department of Chemistry, Bharathiar University, Coimbatore 641 046, India

## Abstract

In the title compound, C_25_H_19_ClN_2_, the dibenzo[*b*,*h*][1,6]naphthyridine system is planar to within 0.16 (2) Å, and the chloro­phenyl ring is inclined to it by 82.53 (7)°. In the crystal, mol­ecules are linked by C—H⋯N hydrogen bonds, forming chains propagating in [100]. There are also a number of weak π–π stacking inter­actions present [centroid–centroid distances = 3.8531 (1) and 3.7631 (1) Å].

## Related literature

For the biological properties of [1,6]naphthyridine derivatives, see: Zhuang *et al.* (2003 ▶); Bedard *et al.* (2003 ▶); Hinschberger *et al.* (2003 ▶); Naik *et al.* (2006 ▶). For the synthesis of the precursor of the title compound, see: Nandha Kumar *et al.* (2007 ▶). For the crystal structures of other naphthrydine derivatives, see: Sivakumar *et al.* (2003 ▶); Fun *et al.* (2009 ▶); Vennila *et al.* (2010 ▶). For standard bond lengths, see: Allen *et al.* (1987 ▶).
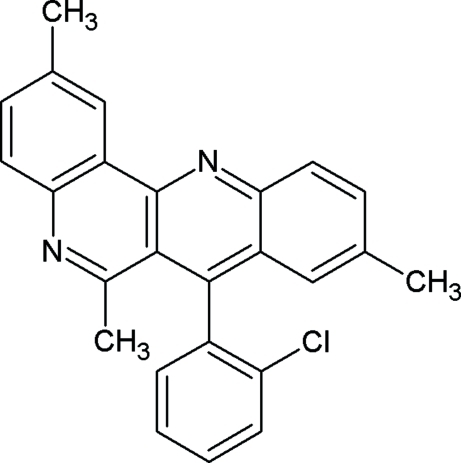


## Experimental

### 

#### Crystal data

C_25_H_19_ClN_2_
*M* *_r_* = 382.87
Triclinic, 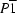

*a* = 6.5575 (4) Å
*b* = 10.6538 (7) Å
*c* = 14.3522 (9) Å
α = 93.755 (3)°
β = 103.099 (3)°
γ = 102.074 (3)°
*V* = 948.25 (10) Å^3^
*Z* = 2
Mo *K*α radiation
μ = 0.21 mm^−1^
*T* = 293 K
0.27 × 0.25 × 0.23 mm

#### Data collection

Bruker SMART APEXII area-detector diffractometer
Absorption correction: multi-scan (*SADABS*; Bruker, 2001 ▶) *T*_min_ = 0.944, *T*_max_ = 0.952
17317 measured reflections
4771 independent reflections
3514 reflections with *I* > 2σ(*I*)
*R*_int_ = 0.025

#### Refinement

*R*[*F* ^2^ > 2σ(*F* ^2^)] = 0.046
*wR*(*F* ^2^) = 0.143
*S* = 1.00
4771 reflections
255 parameters
H-atom parameters constrained
Δρ_max_ = 0.32 e Å^−3^
Δρ_min_ = −0.51 e Å^−3^

### 

Data collection: *APEX2* (Bruker, 2007 ▶); cell refinement: *SAINT* (Bruker, 2007 ▶); data reduction: *SAINT*; program(s) used to solve structure: *SHELXS97* (Sheldrick, 2008 ▶); program(s) used to refine structure: *SHELXL97* (Sheldrick, 2008 ▶); molecular graphics: *ORTEP-3* (Farrugia, 1997 ▶); software used to prepare material for publication: *SHELXL97* and *PLATON* (Spek, 2009 ▶).

## Supplementary Material

Crystal structure: contains datablocks global, I. DOI: 10.1107/S1600536810030576/su2198sup1.cif

Structure factors: contains datablocks I. DOI: 10.1107/S1600536810030576/su2198Isup2.hkl

Additional supplementary materials:  crystallographic information; 3D view; checkCIF report

## Figures and Tables

**Table 1:** Hydrogen-bond geometry (Å, °)

*D*—H⋯*A*	*D*—H	H⋯*A*	*D*⋯*A*	*D*—H⋯*A*
C22—H22⋯N7^i^	0.93	2.42	3.318 (2)	162

Symmetry code: (i) 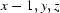.

**Fig. 1.**
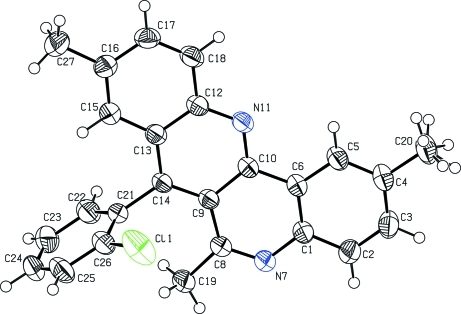
View of the title molecule showing the displacement ellipsoids drawn at 50% probability level.

**Fig. 2.**
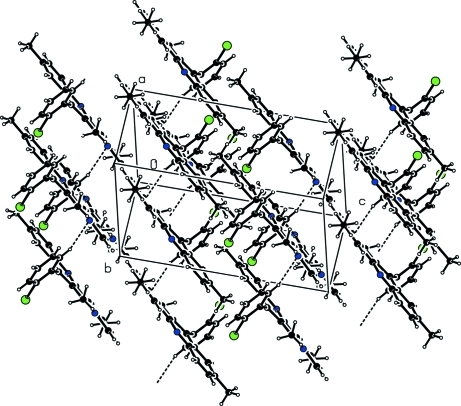
The crystal packing of the title compound illustrating the formation of the C-H···N hydrogen bonded (dashed lines) chain propagating along [100], and the π–π interactions (dashed lines); see Tables 1 and 2 for details.

## supplementary crystallographic information

### Comment

[1,6]naphthyridine derivatives are reported to be a good class of HIV integrase inhibitors (Zhuang *et al.*, 2003). Their antiviral properties have also been reported by (Bedard *et al.*, 2003), and they were proved to be selective antagonists of 5-HT4 receptors (Hinschberger *et al.*, 2003). The planar fused heterocyclic system in the title compound may lead to its DNA intercalating property (Naik *et al.*, 2006). Due to this biological importance, the title compound was chosen for structural studies.

The title compound, illustrated in Fig. 1, consists of a dibenzo[*b*,*h*][1,6]naphthyridine core with chlorophenyl and methyl group substitutions. The bond lengths are in normal ranges (Allen *et al.*, 1987), and are similar to those observed for other naphthrydine derivatives (Sivakumar *et al.*, 2003; Fun *et al.*, 2009; Vennila *et al.*, 2010), as are the bond angles. The dihedral angle between the fused ring dibenzo[*b*,*h*][1,6]naphthyridine system (N1/N2/C1/C6/C8-C10/C12-C14; planar to within 0.16 (2) Å) and the chlorophenyl ring was found to be 82.53 (7)°. There is an apparent steric clash between the methyl group attached to C8 and the chlorophenyl ring attached to C14. The C9-C8-C19 bond angle of 123.05 (13)° and the C9-C14-C21 bond angle of 124.24 (12)°, suggests that these groups are being forced apart.

The crystal packing is stabilized by C—H···N hydrogen bonds forming chain like patterns propagating along [100] (Table 1, Fig. 2). A number of weak π–π stacking interactions may also stablize the crystal packing (see Table 2 for details).

### Experimental

The precursor of the title compoud, 2,6,4'-trimethyl-4-(*N*-phenylamino) quinoline, was prepared following the procedure of (Nandha Kumar *et al.*., 2007). 4-Chloro-2,6-dimethylquinoline (0.002 mol) was reacted with *p*-toluidine (0.002 mol) under neat conditions at 433 K for 30 mins. The product obtained was washed with water, dried, and purified by column chromatography over silica gel using an ethyl acetate:methanol (95:5) mixture to obtain the product as a white solid. A mixture of 2,6,4'-trimethyl-4-(*N*-phenylamino) quinoline (0.001 mol) and *o*-chlorobenzoic acid (0.0011 mol) was added to polyphosphoric acid (1 g of P_2_O_5_ and 0.5 ml H_3_PO_4_) and heated at 433 k for 5 h. The reaction mixture was poured into ice water, neutralized with saturated sodium bicarbonate solution to remove the excess of *o*-chlorobenzoic acid, extracted with ethyl acetate. It was then purified using silica gel column chromatography and the product was eluted with a petroleum ether:ethyl acetate (99:1) mixture to get the final product as a pale yellow solid.  Recrystallization using ethanol gave yellow block-like crystals of the title compound suitable for X-ray diffraction analysis.

### Refinement

The H-atoms of methyl group C20 were disordered over two positions and were placed in calculated positions and treated as riding atoms, each with an occupancy of 0.5. The remaining H-atoms were positioned geometrically and treated as riding on their parent atoms; N—H = 0.86 Å, C—H = 0.93, 0.96 and 0.97 Å, for CH(aromatic), methyl and methylene H-atoms, respectively, with *U*_iso_ = k × *U*_eq_(parent N, or C atom), where k = 1.5 for methyl H-atoms and 1.2 for all other H-atoms.

### Crystal data

**Table:**

C_25_H_19_ClN_2_	*Z* = 2
*M**_r_* = 382.87	*F*(000) = 400
Triclinic, *P*1	*D*_x_ = 1.341 Mg m^−^^3^
Hall symbol: -P 1	Mo *K*α radiation, λ = 0.71073 Å
*a* = 6.5575 (4) Å	Cell parameters from 4797 reflections
*b* = 10.6538 (7) Å	θ = 1.5–28.5°
*c* = 14.3522 (9) Å	µ = 0.21 mm^−^^1^
α = 93.755 (3)°	*T* = 293 K
β = 103.099 (3)°	Block, yellow
γ = 102.074 (3)°	0.27 × 0.25 × 0.23 mm
*V* = 948.25 (10) Å^3^	

### Data collection

**Table:**

Bruker SMART APEXII area-detector diffractometer	4771 independent reflections
Radiation source: fine-focus sealed tube	3514 reflections with *I* > 2σ(*I*)
graphite	*R*_int_ = 0.025
ω and φ scans	θ_max_ = 28.5°, θ_min_ = 1.5°
Absorption correction: multi-scan (*SADABS*; Bruker, 2001)	*h* = −8→8
*T*_min_ = 0.944, *T*_max_ = 0.952	*k* = −14→14
17317 measured reflections	*l* = −19→19

### Refinement

**Table:**

Refinement on *F*^2^	Primary atom site location: structure-invariant direct methods
Least-squares matrix: full	Secondary atom site location: difference Fourier map
*R*[*F*^2^ > 2σ(*F*^2^)] = 0.046	Hydrogen site location: inferred from neighbouring sites
*wR*(*F*^2^) = 0.143	H-atom parameters constrained
*S* = 1.00	*w* = 1/[σ^2^(*F*_o_^2^) + (0.0694*P*)^2^ + 0.2763*P*] where *P* = (*F*_o_^2^ + 2*F*_c_^2^)/3
4771 reflections	(Δ/σ)_max_ = 0.001
255 parameters	Δρ_max_ = 0.32 e Å^−^^3^
0 restraints	Δρ_min_ = −0.51 e Å^−^^3^

### Special details

**Table:**

Geometry. All esds (except the esd in the dihedral angle between two l.s. planes) are estimated using the full covariance matrix. The cell esds are taken into account individually in the estimation of esds in distances, angles and torsion angles; correlations between esds in cell parameters are only used when they are defined by crystal symmetry. An approximate (isotropic) treatment of cell esds is used for estimating esds involving l.s. planes.
Refinement. Refinement of F^2^ against ALL reflections. The weighted R-factor wR and goodness of fit S are based on F^2^, conventional R-factors R are based on F, with F set to zero for negative F^2^. The threshold expression of F^2^ > 2sigma(F^2^) is used only for calculating R-factors(gt) etc. and is not relevant to the choice of reflections for refinement. R-factors based on F^2^ are statistically about twice as large as those based on F, and R- factors based on ALL data will be even larger.

### Fractional atomic coordinates and isotropic or equivalent isotropic displacement parameters (Å^2^)

**Table:**

	*x*	*y*	*z*	*U*_iso_*/*U*_eq_	Occ. (<1)
Cl1	0.22259 (9)	0.49217 (5)	0.41457 (4)	0.0809 (2)	
C9	0.1060 (2)	0.63949 (13)	0.20011 (10)	0.0345 (3)	
C14	−0.0361 (2)	0.61889 (13)	0.25997 (11)	0.0354 (3)	
N11	0.1756 (2)	0.87266 (11)	0.24184 (9)	0.0396 (3)	
C10	0.2149 (2)	0.77063 (13)	0.19709 (10)	0.0353 (3)	
C13	−0.0816 (2)	0.72702 (14)	0.30668 (11)	0.0371 (3)	
C6	0.3810 (2)	0.79371 (14)	0.14465 (10)	0.0367 (3)	
C1	0.4188 (2)	0.68758 (14)	0.09475 (11)	0.0382 (3)	
C12	0.0265 (2)	0.85236 (14)	0.29330 (11)	0.0379 (3)	
N7	0.2977 (2)	0.56271 (12)	0.09035 (9)	0.0405 (3)	
C5	0.5087 (3)	0.91727 (15)	0.14455 (12)	0.0427 (3)	
H5	0.4829	0.9885	0.1770	0.051*	
C21	−0.1424 (2)	0.48827 (13)	0.28004 (11)	0.0383 (3)	
C15	−0.2299 (3)	0.71701 (15)	0.36619 (12)	0.0426 (3)	
H15	−0.2983	0.6356	0.3770	0.051*	
C2	0.5826 (3)	0.70513 (17)	0.04574 (13)	0.0478 (4)	
H2	0.6075	0.6347	0.0119	0.057*	
C18	−0.0219 (3)	0.96186 (15)	0.33756 (12)	0.0459 (4)	
H18	0.0465	1.0444	0.3289	0.055*	
C8	0.1537 (2)	0.53873 (14)	0.13957 (11)	0.0376 (3)	
C16	−0.2740 (3)	0.82394 (16)	0.40769 (12)	0.0435 (4)	
C4	0.6710 (3)	0.93469 (16)	0.09730 (12)	0.0463 (4)	
C26	−0.0401 (3)	0.42446 (15)	0.35054 (12)	0.0450 (4)	
C17	−0.1666 (3)	0.94743 (16)	0.39226 (12)	0.0471 (4)	
H17	−0.1960	1.0206	0.4204	0.057*	
C22	−0.3525 (3)	0.42820 (16)	0.23021 (15)	0.0529 (4)	
H22	−0.4273	0.4693	0.1833	0.063*	
C3	0.7064 (3)	0.82702 (17)	0.04798 (13)	0.0512 (4)	
H3	0.8162	0.8381	0.0160	0.061*	
C23	−0.4512 (3)	0.30820 (18)	0.24972 (17)	0.0622 (5)	
H23	−0.5900	0.2684	0.2146	0.075*	
C19	0.0335 (3)	0.39954 (15)	0.12548 (14)	0.0502 (4)	
H19A	0.0790	0.3520	0.0779	0.075*	
H19B	−0.1181	0.3942	0.1043	0.075*	
H19C	0.0630	0.3635	0.1853	0.075*	
C25	−0.1401 (3)	0.30526 (16)	0.37131 (14)	0.0563 (5)	
H25	−0.0683	0.2646	0.4195	0.068*	
C24	−0.3463 (3)	0.24775 (17)	0.32009 (15)	0.0583 (5)	
H24	−0.4144	0.1676	0.3334	0.070*	
C20	0.8124 (3)	1.06674 (18)	0.10020 (15)	0.0613 (5)	
H20A	0.9161	1.0606	0.0635	0.092*	0.50
H20B	0.8857	1.0999	0.1658	0.092*	0.50
H20C	0.7254	1.1239	0.0732	0.092*	0.50
H20D	0.7687	1.1290	0.1382	0.092*	0.50
H20E	0.7992	1.0897	0.0359	0.092*	0.50
H20F	0.9594	1.0657	0.1284	0.092*	0.50
C27	−0.4337 (3)	0.81378 (19)	0.46916 (15)	0.0603 (5)	
H27A	−0.5716	0.8179	0.4303	0.090*	
H27B	−0.3858	0.8839	0.5203	0.090*	
H27C	−0.4454	0.7331	0.4960	0.090*	

### Atomic displacement parameters (Å^2^)

**Table:**

	*U*^11^	*U*^22^	*U*^33^	*U*^12^	*U*^13^	*U*^23^
Cl1	0.0751 (4)	0.0620 (3)	0.0805 (4)	−0.0032 (2)	−0.0189 (3)	0.0237 (3)
C9	0.0369 (7)	0.0290 (6)	0.0383 (7)	0.0081 (5)	0.0095 (6)	0.0067 (5)
C14	0.0374 (7)	0.0284 (7)	0.0415 (8)	0.0080 (5)	0.0105 (6)	0.0080 (6)
N11	0.0477 (7)	0.0294 (6)	0.0430 (7)	0.0072 (5)	0.0149 (6)	0.0055 (5)
C10	0.0388 (7)	0.0309 (7)	0.0359 (7)	0.0069 (6)	0.0089 (6)	0.0071 (5)
C13	0.0415 (8)	0.0318 (7)	0.0391 (8)	0.0092 (6)	0.0111 (6)	0.0063 (6)
C6	0.0393 (7)	0.0333 (7)	0.0366 (7)	0.0048 (6)	0.0098 (6)	0.0074 (6)
C1	0.0400 (8)	0.0353 (7)	0.0388 (8)	0.0057 (6)	0.0110 (6)	0.0062 (6)
C12	0.0451 (8)	0.0318 (7)	0.0372 (7)	0.0093 (6)	0.0103 (6)	0.0051 (6)
N7	0.0450 (7)	0.0326 (6)	0.0461 (7)	0.0072 (5)	0.0171 (6)	0.0044 (5)
C5	0.0490 (9)	0.0339 (7)	0.0428 (8)	0.0014 (6)	0.0137 (7)	0.0050 (6)
C21	0.0423 (8)	0.0293 (7)	0.0478 (8)	0.0088 (6)	0.0194 (6)	0.0061 (6)
C15	0.0464 (8)	0.0366 (8)	0.0481 (9)	0.0089 (6)	0.0180 (7)	0.0078 (6)
C2	0.0514 (9)	0.0449 (9)	0.0506 (9)	0.0080 (7)	0.0230 (7)	0.0033 (7)
C18	0.0616 (10)	0.0308 (7)	0.0475 (9)	0.0104 (7)	0.0181 (8)	0.0046 (6)
C8	0.0411 (8)	0.0308 (7)	0.0416 (8)	0.0083 (6)	0.0114 (6)	0.0057 (6)
C16	0.0455 (8)	0.0433 (8)	0.0435 (8)	0.0122 (7)	0.0131 (7)	0.0031 (7)
C4	0.0469 (9)	0.0426 (8)	0.0446 (9)	−0.0026 (7)	0.0128 (7)	0.0081 (7)
C26	0.0552 (9)	0.0343 (7)	0.0445 (9)	0.0064 (7)	0.0134 (7)	0.0069 (6)
C17	0.0599 (10)	0.0380 (8)	0.0470 (9)	0.0170 (7)	0.0165 (8)	0.0001 (7)
C22	0.0446 (9)	0.0416 (9)	0.0728 (12)	0.0090 (7)	0.0142 (8)	0.0142 (8)
C3	0.0487 (9)	0.0539 (10)	0.0520 (10)	0.0013 (7)	0.0239 (8)	0.0071 (8)
C23	0.0433 (9)	0.0473 (10)	0.0937 (15)	−0.0002 (8)	0.0221 (10)	0.0087 (10)
C19	0.0589 (10)	0.0321 (7)	0.0617 (11)	0.0036 (7)	0.0268 (8)	−0.0009 (7)
C25	0.0803 (13)	0.0368 (8)	0.0536 (10)	0.0088 (8)	0.0216 (9)	0.0145 (7)
C24	0.0701 (12)	0.0359 (8)	0.0749 (13)	0.0029 (8)	0.0367 (10)	0.0121 (8)
C20	0.0643 (12)	0.0476 (10)	0.0667 (12)	−0.0099 (8)	0.0265 (10)	0.0076 (9)
C27	0.0630 (11)	0.0554 (11)	0.0694 (12)	0.0128 (9)	0.0334 (10)	−0.0011 (9)

### Geometric parameters (Å, °)

**Table:**

Cl1—C26	1.7342 (18)	C8—C19	1.504 (2)
C9—C14	1.399 (2)	C16—C17	1.413 (2)
C9—C10	1.4381 (19)	C16—C27	1.507 (2)
C9—C8	1.465 (2)	C4—C3	1.397 (2)
C14—C13	1.414 (2)	C4—C20	1.507 (2)
C14—C21	1.4978 (19)	C26—C25	1.386 (2)
N11—C10	1.3272 (18)	C17—H17	0.9300
N11—C12	1.3449 (19)	C22—C23	1.384 (2)
C10—C6	1.448 (2)	C22—H22	0.9300
C13—C12	1.422 (2)	C3—H3	0.9300
C13—C15	1.425 (2)	C23—C24	1.365 (3)
C6—C1	1.395 (2)	C23—H23	0.9300
C6—C5	1.405 (2)	C19—H19A	0.9600
C1—N7	1.3888 (19)	C19—H19B	0.9600
C1—C2	1.399 (2)	C19—H19C	0.9600
C12—C18	1.419 (2)	C25—C24	1.374 (3)
N7—C8	1.2966 (19)	C25—H25	0.9300
C5—C4	1.374 (2)	C24—H24	0.9300
C5—H5	0.9300	C20—H20A	0.9600
C21—C26	1.382 (2)	C20—H20B	0.9600
C21—C22	1.394 (2)	C20—H20C	0.9600
C15—C16	1.365 (2)	C20—H20D	0.9600
C15—H15	0.9300	C20—H20E	0.9600
C2—C3	1.374 (2)	C20—H20F	0.9600
C2—H2	0.9300	C27—H27A	0.9600
C18—C17	1.353 (2)	C27—H27B	0.9600
C18—H18	0.9300	C27—H27C	0.9600
			
C14—C9—C10	117.59 (12)	C18—C17—H17	119.2
C14—C9—C8	125.62 (13)	C16—C17—H17	119.2
C10—C9—C8	116.79 (13)	C23—C22—C21	120.83 (17)
C9—C14—C13	118.93 (13)	C23—C22—H22	119.6
C9—C14—C21	124.24 (12)	C21—C22—H22	119.6
C13—C14—C21	116.82 (13)	C2—C3—C4	121.42 (15)
C10—N11—C12	118.23 (12)	C2—C3—H3	119.3
N11—C10—C9	123.65 (13)	C4—C3—H3	119.3
N11—C10—C6	117.60 (13)	C24—C23—C22	120.50 (18)
C9—C10—C6	118.73 (12)	C24—C23—H23	119.8
C14—C13—C12	118.07 (13)	C22—C23—H23	119.8
C14—C13—C15	123.54 (13)	C8—C19—H19A	109.5
C12—C13—C15	118.39 (13)	C8—C19—H19B	109.5
C1—C6—C5	119.07 (14)	H19A—C19—H19B	109.5
C1—C6—C10	118.10 (13)	C8—C19—H19C	109.5
C5—C6—C10	122.80 (14)	H19A—C19—H19C	109.5
N7—C1—C6	122.51 (13)	H19B—C19—H19C	109.5
N7—C1—C2	117.55 (13)	C24—C25—C26	119.42 (17)
C6—C1—C2	119.94 (14)	C24—C25—H25	120.3
N11—C12—C18	118.06 (13)	C26—C25—H25	120.3
N11—C12—C13	123.19 (13)	C23—C24—C25	120.02 (16)
C18—C12—C13	118.73 (14)	C23—C24—H24	120.0
C8—N7—C1	120.50 (13)	C25—C24—H24	120.0
C4—C5—C6	121.15 (15)	C4—C20—H20A	109.5
C4—C5—H5	119.4	C4—C20—H20B	109.5
C6—C5—H5	119.4	H20A—C20—H20B	109.5
C26—C21—C22	117.31 (14)	C4—C20—H20C	109.5
C26—C21—C14	121.67 (14)	H20A—C20—H20C	109.5
C22—C21—C14	120.98 (14)	H20B—C20—H20C	109.5
C16—C15—C13	121.68 (15)	C4—C20—H20D	109.5
C16—C15—H15	119.2	H20A—C20—H20D	141.1
C13—C15—H15	119.2	H20B—C20—H20D	56.3
C3—C2—C1	119.59 (15)	H20C—C20—H20D	56.3
C3—C2—H2	120.2	C4—C20—H20E	109.5
C1—C2—H2	120.2	H20A—C20—H20E	56.3
C17—C18—C12	120.70 (15)	H20B—C20—H20E	141.1
C17—C18—H18	119.6	H20C—C20—H20E	56.3
C12—C18—H18	119.6	H20D—C20—H20E	109.5
N7—C8—C9	122.88 (13)	C4—C20—H20F	109.5
N7—C8—C19	114.02 (13)	H20A—C20—H20F	56.3
C9—C8—C19	123.05 (13)	H20B—C20—H20F	56.3
C15—C16—C17	118.83 (15)	H20C—C20—H20F	141.1
C15—C16—C27	121.87 (15)	H20D—C20—H20F	109.5
C17—C16—C27	119.31 (14)	H20E—C20—H20F	109.5
C5—C4—C3	118.82 (15)	C16—C27—H27A	109.5
C5—C4—C20	120.96 (16)	C16—C27—H27B	109.5
C3—C4—C20	120.20 (15)	H27A—C27—H27B	109.5
C21—C26—C25	121.90 (16)	C16—C27—H27C	109.5
C21—C26—Cl1	119.98 (12)	H27A—C27—H27C	109.5
C25—C26—Cl1	118.11 (14)	H27B—C27—H27C	109.5
C18—C17—C16	121.65 (14)		
			
C10—C9—C14—C13	6.1 (2)	C13—C14—C21—C22	82.85 (19)
C8—C9—C14—C13	−173.70 (13)	C14—C13—C15—C16	−178.02 (15)
C10—C9—C14—C21	−172.70 (13)	C12—C13—C15—C16	1.9 (2)
C8—C9—C14—C21	7.5 (2)	N7—C1—C2—C3	179.93 (16)
C12—N11—C10—C9	0.4 (2)	C6—C1—C2—C3	0.6 (3)
C12—N11—C10—C6	−177.97 (13)	N11—C12—C18—C17	−178.30 (15)
C14—C9—C10—N11	−5.4 (2)	C13—C12—C18—C17	0.6 (2)
C8—C9—C10—N11	174.47 (13)	C1—N7—C8—C9	1.4 (2)
C14—C9—C10—C6	172.95 (13)	C1—N7—C8—C19	178.83 (14)
C8—C9—C10—C6	−7.2 (2)	C14—C9—C8—N7	−175.27 (14)
C9—C14—C13—C12	−2.4 (2)	C10—C9—C8—N7	4.9 (2)
C21—C14—C13—C12	176.51 (13)	C14—C9—C8—C19	7.5 (2)
C9—C14—C13—C15	177.54 (14)	C10—C9—C8—C19	−172.28 (14)
C21—C14—C13—C15	−3.6 (2)	C13—C15—C16—C17	−1.1 (2)
N11—C10—C6—C1	−177.95 (13)	C13—C15—C16—C27	178.85 (16)
C9—C10—C6—C1	3.6 (2)	C6—C5—C4—C3	0.6 (3)
N11—C10—C6—C5	4.1 (2)	C6—C5—C4—C20	−177.91 (16)
C9—C10—C6—C5	−174.35 (14)	C22—C21—C26—C25	0.3 (2)
C5—C6—C1—N7	−179.11 (14)	C14—C21—C26—C25	178.09 (15)
C10—C6—C1—N7	2.8 (2)	C22—C21—C26—Cl1	179.19 (13)
C5—C6—C1—C2	0.1 (2)	C14—C21—C26—Cl1	−3.0 (2)
C10—C6—C1—C2	−177.91 (14)	C12—C18—C17—C16	0.2 (3)
C10—N11—C12—C18	−177.28 (14)	C15—C16—C17—C18	0.0 (3)
C10—N11—C12—C13	3.8 (2)	C27—C16—C17—C18	−179.91 (17)
C14—C13—C12—N11	−2.8 (2)	C26—C21—C22—C23	−1.4 (3)
C15—C13—C12—N11	177.25 (14)	C14—C21—C22—C23	−179.24 (16)
C14—C13—C12—C18	178.29 (14)	C1—C2—C3—C4	−0.8 (3)
C15—C13—C12—C18	−1.6 (2)	C5—C4—C3—C2	0.2 (3)
C6—C1—N7—C8	−5.5 (2)	C20—C4—C3—C2	178.72 (18)
C2—C1—N7—C8	175.23 (15)	C21—C22—C23—C24	1.7 (3)
C1—C6—C5—C4	−0.8 (2)	C21—C26—C25—C24	0.6 (3)
C10—C6—C5—C4	177.17 (14)	Cl1—C26—C25—C24	−178.38 (14)
C9—C14—C21—C26	83.9 (2)	C22—C23—C24—C25	−0.9 (3)
C13—C14—C21—C26	−94.89 (17)	C26—C25—C24—C23	−0.3 (3)
C9—C14—C21—C22	−98.31 (19)		

### Hydrogen-bond geometry (Å, °)

**Table:**

*D*—H···*A*	*D*—H	H···*A*	*D*···*A*	*D*—H···*A*
C22—H22···N7^i^	0.93	2.42	3.318 (2)	162

Symmetry codes: (i) *x*−1, *y*, *z*.

### Table 2 π–π interactions [Å] Cg2 is centroid of ring N11,C9,C10,C12-C14; Cg3 is centroid of ring C1-C6; Cg4 is centroid of ring C12,C13,C15-C18. Symmetry codes : (i) -1+x, y,z; (ii) 1+x, y,z.

**Table:**

Cg(I)	Cg(J)	Centroid-to-Centroid
Cg(2)	Cg(3^i^)	3.8531 (1)
Cg(3)	Cg(2^ii^)	3.8531 (1)
Cg(3)	Cg(4^ii^)	3.7631 (1)
Cg(4)	Cg(3^i^)	3.7631 (1)

## References

[bb1] Allen, F. H., Kennard, O., Watson, D. G., Brammer, L., Orpen, A. G. & Taylor, R. (1987). *J. Chem. Soc. Perkin Trans. 2*, pp. S1–19.

[bb2] Bedard, J. S., Lucille, L. H., Thomas, S., Alice, C., John, D., John, H., Laval, C., Haolun, J. & Robert, F. R. (2003). *Antimicrob. Agents Chemother.* **44**, 929–937.

[bb3] Bruker (2001). *SADABS* Bruker AXS Inc., Madison, Wisconsin, USA.

[bb4] Bruker (2007). *APEX2* and *SAINT* Bruker AXS Inc., Madison, Wisconsin, USA.

[bb5] Farrugia, L. J. (1997). *J. Appl. Cryst.* **30**, 565.

[bb6] Fun, H.-K., Yeap, C. S., Das, N. K., Mahapatra, A. K. & Goswami, S. (2009). *Acta Cryst.* E**65**, o1747. 10.1107/S1600536809024350 PMC2977386 21583458

[bb7] Hinschberger, A., Butt, S., Lelong, V., Boulouard, M., Dumuis, A., Dauphin, F., Bureau, R., Pfeiffer, B., Renard, P. & Rault, S. (2003). *J. Med. Chem.* **46**, 138–147. 10.1021/jm020954v 12502367

[bb8] Naik, T. R., Naik, S. H., Raghavendra, M. & Naik, S. G. K. (2006). *ARKIVOC*, **xv**, 84–94.

[bb9] Nandha Kumar, R., Suresh, T., Dhanabal, T. & Mohan, P. S. (2007). *Indian J. Chem. Sect B.* **46**, 995–1000.

[bb10] Sheldrick, G. M. (2008). *Acta Cryst.* A**64**, 112–122. 10.1107/S0108767307043930 18156677

[bb11] Sivakumar, B., SethuSankar, K., Senthil Kumar, U. P., Jeyaraman, R. & Velmurugan, D. (2003). *Acta Cryst.* C**59**, o153–o155. 10.1107/s0108270102022758 12711793

[bb12] Spek, A. L. (2009). *Acta Cryst.* D**65**, 148–155. 10.1107/S090744490804362X PMC2631630 19171970

[bb13] Vennila, K. N., Prabha, K., Manoj, M., Prasad, K. J. R. & Velmurugan, D. (2010). *Acta Cryst.* E**66**, o1823. 10.1107/S160053681002430X PMC3007064 21588030

[bb14] Zhuang, L., Wai, J. S., Embrey, W. M., Fisher, E. T., Egbertson, S. M., Payne, S. L., Guare, P. J., Vacca, P. J., Hazuda, J. D., Felock, J. P., Wolfe, L. A., Stillmock, A. K., Witmer, V. M., Moyer, G., Schleif, A. W., Gabryelski, J. L., Leonard, M. Y., Lynch, J. J., Michelson, R. S. & Young, D. S. (2003). *J. Med. Chem.* **46**, 453–456.

